# Posterior Rectus Sheath Defect with Interparietal Hernia Causing a Small Bowel Obstruction

**DOI:** 10.1155/2022/2616381

**Published:** 2022-04-05

**Authors:** Austin Vegas, Caroline Shea, Paul Sparzak

**Affiliations:** ^1^Campbell University School of Osteopathic Medicine, 4350 US-421 Lillington, NC 27546, USA; ^2^Department of OBGYN, Cape Fear Valley Medical Center, 1638 Owen Drive, Fayetteville, NC 28304, USA

## Abstract

Posterior rectus sheath hernias are exceptionally rare, with around twelve reported cases to date. This case report examines a 38-year-old female who demonstrated symptoms of intermittent small bowel obstruction five days following an abdominal hysterectomy. The patient was diagnosed via CT to have a small bowel obstruction within the rectus abdominis. Exploratory laparotomy determined the etiology to be an interparietal hernia through a posterior rectus wall defect, which was repaired with primary closure. Postoperatively, the patient was again unable to tolerate food. Repeat CT showed concern for repeat SBO, though symptoms subsided without intervention. The patient had no complaints during her follow up at one month. This report was aimed at building upon the few reported cases as well as enumerating potential risk factors that may allow for the consideration of this diagnosis in the future.

## 1. Introduction

Hernias are any abnormal protrusion of an organ or tissue through the wall of the cavity containing it [1]. Hernias may be either congenital or acquired, with the most common locations being ventral, femoral, inguinal, and incisional. Less common are interparietal hernias, which occur when the hernial sac lies within the muscles of the abdominal wall but does not enter the subcutaneous tissue [2]. A posterior rectus sheath hernia is a subtype of interparietal hernia and has only been documented twelve times from 1937 to 2019 [1–7].

This report describes the case of a 38-year-old female who developed a small bowel obstruction secondary to an interparietal hernia through a posterior rectus sheath defect. We will discuss the initial presentation, management, and potential causes of this case. Informed consent was obtained from the patient to submit this case.

## 2. Case Presentation

A 38-year-old African American female with a past medical history significant for hyperthyroidism and hypertension presented five days postoperatively from an abdominal hysterectomy and lysis of adhesions with acute severe abdominal pain. In the days prior, the patient tolerated food intermittently with sporadic episodes of nausea, vomiting, and mild abdominal pain. She consistently reported passing flatus as well as multiple loose bowel movements since the surgery. The patient was noncompliant with medication at home but was placed on her original home dose of methimazole and losartan-hydrochlorothiazide while hospitalized.

A review of the patient's surgical history revealed the abdominal hysterectomy was performed through the prior cesarean section scar with a Pfannenstiel entry but was converted intraoperatively to a partial Maylard due to exposure difficulties. Consequently, this method of entry involved incising the rectus abdominal musculature. The incision was closed using mass closure and a malleable retractor for bowel protection.

**Figure 1:**
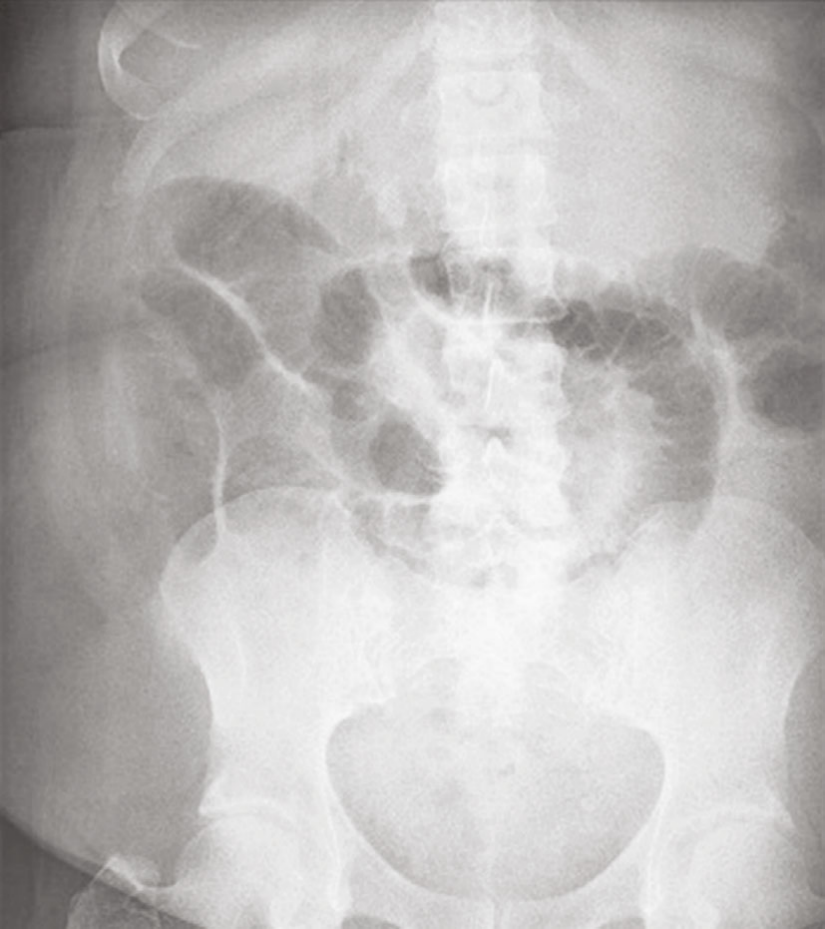
AP supine X-ray of the abdomen showing moderate gaseous distention of the small bowel.

**Figure 2:**
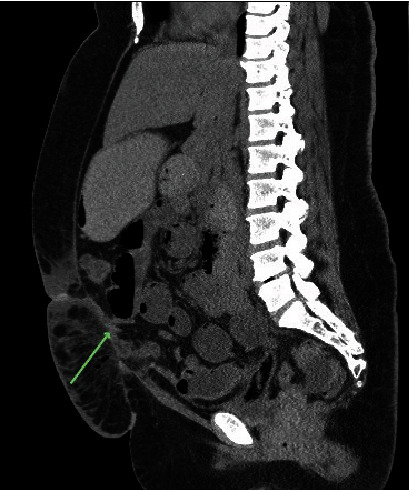
CT abdomen and pelvis showing a markedly distended stomach and proximal jejunum with a transition zone within the rectus abdominis musculature.

Due to the variability of the patient's symptoms, an abdominal X-ray was ordered [Figure 1]. Imaging showed moderate gaseous distention of the small bowel, consistent with small bowel obstruction or ileus. Computed Tomography (CT) abdomen and pelvis was performed to distinguish between ileus and small bowel obstruction [Figure 2]. This revealed a markedly distended stomach and proximal jejunum with a transition zone within the rectus abdominis musculature.

The patient's intermittent tolerance of food, intermittent nausea and vomiting, ability to pass flatus and stool, and recent operation yielded a broad differential. These included postoperative pain, overtreatment of hyperthyroidism, and postoperative ileus. CT revealed small bowel within the abdominal wall musculature, a differential diagnosis of small bowel obstruction was considered due to dehiscence, spigelian hernia, surgical error, or a posterior fascial defect. The small bowel obstruction was treated with emergent exploratory laparotomy which revealed a well-approximated anterior fascia. Sutures were removed and the presence of the small bowel within the posterior rectus sheath defect was confirmed, and an interparietal hernia diagnosis was made. The small bowel was reduced, and primary layered closure was performed without complication.

The patient remained NPO with a nasogastric (NG) tube in place for two days until flatus was passed. She tolerated a clear liquid diet without nausea or vomiting upon NG tube removal. However, the patient experienced nausea with approximately two liters of subsequent bilious emesis five days postoperatively. Abdominal X-ray showed dilated loops of the small bowel, concerning for repeat small bowel obstruction or a focal ileus. Repeat CT revealed a dilated stomach and small bowel with concern for continued intermittent small bowel obstruction. The patient refused the placement of an NG tube and thus was made NPO. On day seven, the patient tolerated food again with resolution of the nausea and vomiting. Patient was discharge home in stable condition. Follow up in the outpatient office was uneventful with patient doing well.

## 3. Discussion

Over one million hernia surgeries are performed in the United States annually, making hernia repairs one of the most common procedures performed [8]. Of these one million surgeries, interparietal hernias constitute less than 2% [9, 10]. Posterior rectus sheath hernias are a subset of the interparietal hernias, with the majority occurring post surgically or post traumatically and a small minority reported as congenital cases [1]. There have been twelve reports of posterior rectus sheath hernia cases among all three categories. Most cases presented with a small bowel obstruction; however, our case differed in postsurgical presentation. It is difficult to determine if this occurred as a result of an isolated posterior dehiscence, prior defect, or spontaneous herniation.

Due to the rarity of this diagnosis, not much is understood about its pathophysiology. Anatomically, the rectus sheath encloses the rectus abdominis muscle. The contents and strength of this barrier depend on its location in relation to the arcuate line. Superior to this line, herniation is rarely seen, as here the posterior sheath is comprised of transversalis fascia and the aponeurosis of the transversus abdominis, internal oblique, and external oblique. In contrast, there is greater concern for herniation inferior to the arcuate line proven by biomechanical testing [11]. This is because the posterior sheath consists of only the transversalis fascia due to all three aponeuroses passing anteriorly to the rectus abdominis muscle [7].

It has been hypothesized that any condition which causes muscle weakness or increased intraabdominal pressure, such as pregnancy, obesity, or ascites, may increase the likelihood of posterior rectus sheath hernias [5]. This is critical in considering the possibility of this hernia being secondary to a prior defect or weakness which was made worse after the most recent surgery as opposed to a dehiscence. Our patient was at an increased risk due to her obesity, obstetrical history of Gravida 5 Para 2, surgical history of a cesarean section, and a 20-week sized uterus due to multiple uterine leiomyomas for an unknown duration. It is possible that the patient's risk factors for increased abdominal pressure caused the fascial defect observed, though the patient's sudden small bowel herniation was likely due to muscle weakness secondary to the rectus abdominis incision.

The literature suggests that treatment of posterior rectus sheath hernias is straightforward regardless of the herniation contents [7]. Within the twelve recorded cases, three were treated nonoperatively, one was treated laparoscopically with a mesh repair, and the rest were treated with primary closure [1–7]. Prosthetic repair may be applicable in some elective cases as well as with larger hernias, but in cases of obstruction, there is an increased risk of bacterial contamination [2, 7].

In conclusion, a woman had a spontaneous small bowel obstruction through a posterior rectus sheath herniation following an abdominal hysterectomy, the patient was managed operatively with a successful outcome. The patient had several risk factors for development of a fascial defect which may have predisposed this patient, though she never had any symptoms prior to the surgery. Although posterior rectus sheath hernias are rare, they should be considered in patients with a history of prolonged intraabdominal pressure, evidence of muscle weakness, or prior abdominal surgeries. This case report was aimed at enumerating potential risk factors that may allow for the consideration of this diagnosis in the future.
